# Anna: an open-source platform for real-time integration of machine learning classifiers with veterinary electronic health records

**DOI:** 10.1186/s12917-025-05000-7

**Published:** 2025-10-02

**Authors:** Chun Yin Kong, Picasso Vasquez, Makan Farhoodimoghadam, Chris Brandt, Titus C. Brown, Krystle L. Reagan, Allison Zwingenberger, Stefan M. Keller

**Affiliations:** 1 Department of Pathology, Microbiology, Immunology, University of California Davis, Davis, CA USA; 2 Independent Researcher, San Francisco, CA USA; 3 Department of Computer Science, University of California Davis, Davis, CA USA; 4 School of Veterinary Medicine Information Technology, University of California Davis, Davis, CA USA; 5 Department of Population Health & Reproduction, University of California Davis, Davis, CA USA; 6 Department of Clinical Sciences, College of Veterinary Medicine and Biomedical Sciences, Colorado State University, Fort Collins, CO 80523 USA; 7 Department of Surgical & Radiological Sciences, University of California Davis, Davis, CA USA

**Keywords:** Electronic health records, Machine learning, Machine learning classifiers, Machine learning integration, Real-time data analysis, Leptospirosis, Hypoadrenocorticism, Portosystemic shunt, Veterinary medicine, Artificial intelligence, Clinical decision-making support

## Abstract

**Background:**

In the rapidly evolving landscape of veterinary healthcare, integrating machine learning (ML) clinical decision-making tools with electronic health records (EHRs) promises to improve diagnostic accuracy and patient care. However, the seamless integration of ML classifiers into existing EHR systems in veterinary medicine is often hindered by the inherent rigidity of these systems or by the limited availability of IT resources to implement the modifications necessary for ML compatibility.

**Results:**

Anna is a standalone analytics platform that can host ML classifiers and interfaces with EHR systems to provide classifier predictions for laboratory data in real-time. Following a request from the EHR system, Anna retrieves patient-specific data from the EHR system, merges diagnostic test results based on user-defined temporal criteria and returns predictions for all available classifiers for display in real-time. Anna was developed in Python and is freely available. Because Anna is a stand-alone platform, it does not require substantial modifications to the existing EHR, allowing for easy integration into existing computing infrastructure. To demonstrate Anna’s versatility, we implemented three previously published ML classifiers to predict a diagnosis of hypoadrenocorticism, leptospirosis, or a portosystemic shunt in dogs.

**Conclusion:**

Anna is an open-source tool designed to improve the accessibility of ML classifiers for the veterinary community. Its flexible architecture supports the integration of classifiers developed in various programming languages and with diverse environment requirements. Anna facilitates rapid prototyping, enabling researchers and developers to deploy ML classifiers quickly without modifications to the existing EHR system. Anna could drive broader adoption of ML in veterinary practices, ultimately enhancing diagnostic capabilities and patient outcomes.

**Supplementary Information:**

The online version contains supplementary material available at 10.1186/s12917-025-05000-7.

## Background

Artificial intelligence and machine learning (ML) have become increasingly popular in aiding clinical decision-making in veterinary medicine [1]. Various ML classifiers have been developed to predict the diagnosis of specific diseases based on laboratory data, such as hyperadrenocorticism [2], hypoadrenocorticism [3], leptospirosis [4] and portosystemic shunt [5] in dogs. Despite their availability, implementing existing veterinary ML classifiers in clinical practice faces several hurdles. Classifiers without a web-based graphical user interface (GUI) often require specific software environments and dependencies, such as particular versions of programming languages (e.g., Python or R), libraries (e.g., TensorFlow, PyTorch, or scikit-learn), and supporting tools (e.g., Docker, virtual environments, or package managers like pip or conda). These requirements must be correctly installed and configured on the user's local machine or server (Supplementary Table 1). Veterinarians without advanced computational skills may find installing and configuring the necessary software technically challenging, creating a barrier to adoption [6]. ML classifiers with a GUI alleviate these concerns, providing quick access, more accessible human–computer interactions, and greater user exposure, particularly when web-based. However, GUIs often require users to manually input many clinical parameters, which is effort-intensive and prone to human error. In addition, users must ensure that the units and format of the input data align with the requirements of the ML classifier, which might require value transformations, like grouping of continuous variables, converting concentration units, or standardizing breed information. Another potential limitation of classifiers, whether they employ a GUI or not, is that they rely on manual activation. If a clinician does not consider a disease, the relevant ML classifier will not be triggered, increasing the risk that the condition goes unrecognized. Finally, running multiple classifiers on patient data requires separate, independent analyses for each ML classifier. Consequently, there is a pressing need for a computing environment that automates data analysis with ML classifiers. The analysis should seamlessly integrate with existing electronic health record (EHR) workflows and operate in the background without disrupting the clinician's routine. Crucially, this environment must be user-friendly, requiring no computer skills or specialized knowledge from the clinician.

To overcome these shortfalls, we developed Anna, an analytics tool that facilitates the integration of multiple ML classifiers into a single platform. Anna can retrieve patient data from an EHR system and generates ML classifier predictions without the need for manual data entry. This provides clinicians with just-in-time access to multiple prediction results. We demonstrate Anna’s versatility by integrating three previously published ML classifiers for prediction of hypoadrenocorticism, leptospirosis and portosystemic shunt diagnosis in dogs. Anna is designed with a modular architecture, enabling the integration of future classifiers regardless of the programming languages used.

## Implementation

While Anna is intended to support clinical decision-making by veterinary practitioners, this manuscript focuses on the technical framework required for its successful deployment and integration. Specifically, it is directed at stakeholders responsible for implementing ML infrastructure within veterinary institutions and practice networks—such as administrators, medical informaticians, and IT professionals. Furthermore, this manuscript does not reassess the clinical value of previously described classifiers. Rather, it addresses the technical barrier to their use—namely, the lack of infrastructure to deliver ML predictions directly to the EHR without manual input or technical expertise. We intentionally avoid generalizing about clinical impact, as this depends on factors such as classifier choice, practice type, and patient population. Anna’s modular design allows institutions to integrate the models most relevant to their context. Our focus is on enabling that integration—not prescribing how it will enhance clinical workflows.

### General architecture and software requirements

**Fig. 1:**
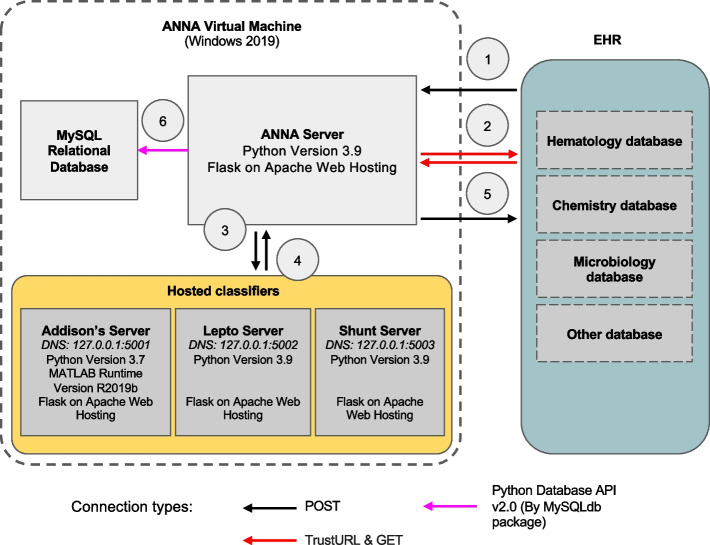
Anna architecture and workflow. The prediction pipeline is initiated when a user accesses a specific test result in the electronic health record (EHR) and includes the following steps: 1) Classifier results request. The EHR sends a request to the Anna, including the patient ID and test date; 2) Data fetch. Anna retrieves relevant diagnostic test results from the EHR; 3) Data pre-processing. Anna merges results from different diagnostic tests according to user-defined temporal criteria and forwards the compiled data to the classifier servers; 4) Classifier servers generate prediction results and return the results to the Anna server; 5) The Anna server passes the classifier results back to the EHR system for display to the end-user; 6) Anna stores classifier results, along with associated timestamps, in a MySQL database. To avoid potential variability in the established performance of classifiers, we match the software of each classifier server to the software versions that the model was developed under

Anna is a stand-alone platform that can host multiple ML classifiers and interfaces with EHRs to provide classifier predictions for patient data in real time. Upon request from an EHR system, Anna fetches relevant laboratory test results from the EHRs, runs available classifiers, and returns classifier prediction results to the EHR system for display to the user (Fig. 1).

Anna is a Python based server built with the Flask web framework and hosted using Apache HTTP Server services. It is a REST server that is not accessible via web browser and doesn’t have a graphical user interface (GUI). Anna is contained in a virtual machine running on Windows Server 2019 and communicates with the EHR system via REST API, an application programming interface that is used to access a resource within another service or application with HTTP requests. The software packages used in Anna are open-source and Anna’s source code and instructions are publicly available (https://github.com/ucdavis/ANNA-AnimalHealthAnalytics).

Machine learning classifiers are hosted in dedicated environments on separate Apache servers and communicate with the Anna core server through unique URL paths. This architecture allows preserving the software versions under which a ML classifier was developed while keeping the Anna core server up to date with respect to securities standards and bug fixes.

To demonstrate Anna’s versatility, we integrated three previously published ML classifiers, each with distinct computational environment requirements: 1) “Addison’s”, a MATLAB-based ML classifier with a Python wrapper script, that employs adaptive boosting with decision trees for the prediction of hypoadrenocorticism in dogs [3], 2) “Lepto”, a Python-based support vector machine (SVM) ML classifier for the prediction of leptospirosis in dogs [4], and 3) “Shunt”, a Python-based extreme gradient boosting (XGBoost) ML classifier for the prediction of portosystemic shunt (PSS) in dogs [5]. All classifiers use at least two of the following data types: signalment, complete blood count, serum chemistry panel, urinalysis, leptospirosis testing request.

Several software libraries are essential for hosting these third-party classifiers on Anna. In general, Anna uses the Python data science package “pandas” [7] to handle data conversions from the EHR during data preprocessing. The Addison’s classifier requires the MATLAB runtime [8], a standalone set of instructions that can execute compiled MATLAB applications or components, to establish connections between Python and MATLAB, and execute MATLAB scripts in Python environments. The advantage of using the MATLAB runtime is that it doesn’t require an active MATLAB license, and it is readily available for download. The leptospirosis classifier utilizes Python data science packages such as “scikit-learn” [9], and “numpy” [10] for further data preprocessing, calculations, and retrieving machine learning prediction results. The shunt classifier requires the “XGBoost” [11] Python package for loading the pre-trained model and generating predictions.

### ML classifier prediction workflow

The classifier prediction pipeline consists of six stages: 1) classifier result request, 2) data fetch, 3) data preprocessing, 4) classifier prediction, 5) result and error reporting, and 6) results storage.

#### Request for classifier result

The generation of ML classifier results is initiated by a REST API request from the EHR system to Anna, containing the patient identification number and query date encapsulated in JSON format. Each classifier is associated with a unique URL. Triggers for initiating a request are flexible and may include a user activating a specific control or merely viewing a particular test result. In the current implementation, the trigger is viewing any laboratory test results that is required for a classifier where the patient species is “Canine” and the user is authorized to have access to the ML analysis.

#### Data fetch

**Fig. 2:**
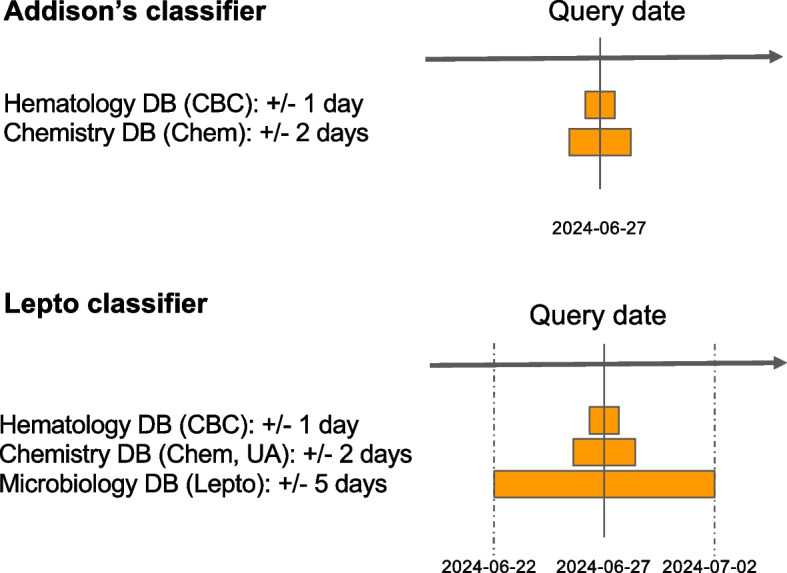
Considerations for time range selection when retrieving data from the EHR. To capture test results reported on days other than the query date, users can specify query time ranges for each database (DB) and classifier. The query date serves as the reference, and results are retrieved for the designated period before and after this date. For example: If the test date is “2024-06-27” (YYYY-MM-DD), the following date ranges will be fetched: Hematology DB: 2024-06-26 to 2024-06-28, Chemistry DB: 2024-06-25 to 2024-05-29, and Microbiology 2024-06-22 to 2024-07-02

To retain a lean architecture and minimize the chances of data loss, Anna only stores classifier results but no original patient data. Consequently, patient data is fetched from the EHR for each request. In the current implementation, each data fetch queries several EHR database tables (e.g. hematology, chemistry, microbiology, etc.) in parallel. The use of parallelism for data fetching significantly accelerates the execution of multiple queries as it is an I/O bound process. The EHR supports a REST-based process to request laboratory data and receive results in XML format when provided with a patient ID, date range, and laboratory section of interest. Access to this interface is restricted to specific IP addresses and requires an authorization code in order to respond to the request. Given that classifiers commonly depend on more than one type of laboratory test (e.g. CBC, serum chemistry panel and urinalysis) that may become available over several days, it is pertinent to capture test results that are reported in close temporal proximity. Anna fetches laboratory results within a user-defined time range from the query date. This time range is specific for each classifier and test type (Fig. 2).

#### Data preprocessing

**Fig. 3:**
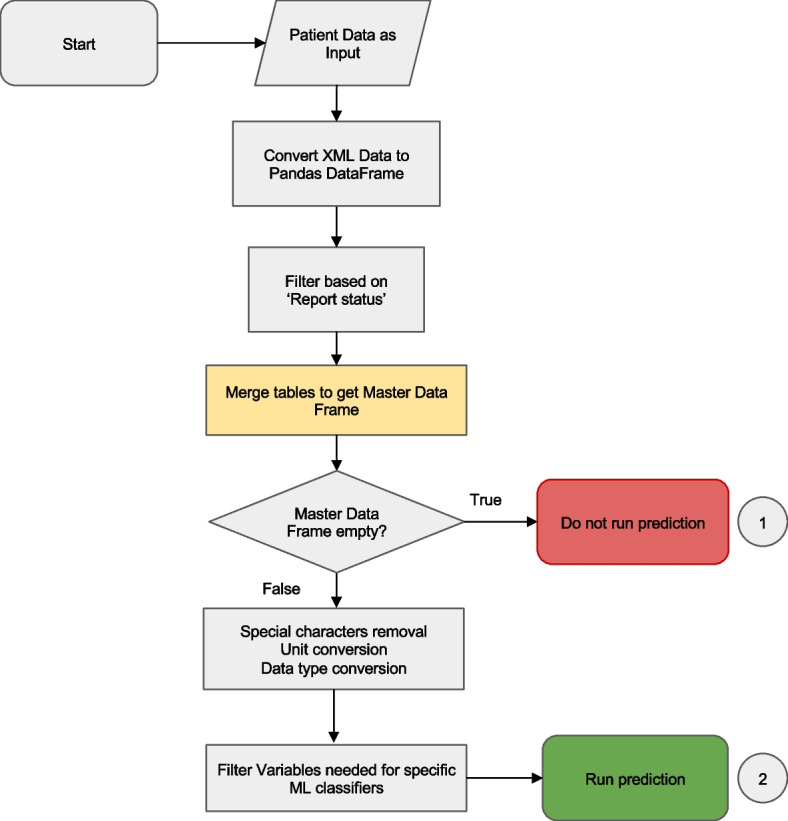
Data preprocessing workflow. The preprocessing merges tables fetched from several databases into a single data frame and performs filtering, data cleaning and conversion functions. If there is insufficient data, the classifier is not run and a result code of “−1” is returned to the EHR. If there is sufficient data, the master data frame is passed to the classifier server. Details of the ‘table merging’ step (highlighted in yellow) are described in Fig. 4

The preprocessing step cleans data fetched from the EHRs system, merges individual tables into a master data frame and produces a classifier-specific data frame that is then passed to the ML classifier (Fig. 3).

**Fig. 4:**
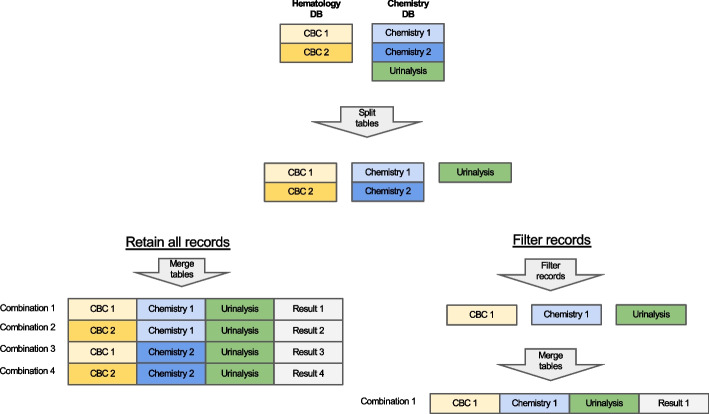
Merging of test results. If the data retrieved from a single database contains multiple test types (e.g. chemistry panel and urinalysis fetched from the Chemistry DB), then each test type is separated into its own table. Tables are then merged in an ‘all-vs-all’ fashion, i.e. if more than one result is available for a given test, then all possible test combinations are generated. For example, for a dataset with two complete blood counts (CBCs) and two chemistry panels (Chemistry), four unique combinations are created, and classifier results are generated for each combination. Alternatively, records can be filtered before table merging to limit the number of combinations generated based on a user-defined rule (e.g. only retain the first test per test type)

First, Anna filters records based on the report status. Typically, a finalized test result is preferred to ensure the accuracy and reliability of results that are passed to a ML classifier. However, in some instances, the test request, rather than the result, is of significance. For instance, the Lepto classifier is applied only when a diagnostic test for leptospirosis, such as Leptospira spp. PCR or microscopic agglutination test has been requested, as it was validated on suspected leptospirosis cases. Consequently, records containing either a finalized Lepto result or simply a test request are retained. Next, Anna merges the data, that were fetched and stored as separate tables, into a single master data frame. The merging logic is explained in Fig. 4.

Subsequently, any special characters and string text, such as those found in comments, are eliminated from the numerical fields followed by the conversion of units (i.e. mg to μg) and data types (e.g. categorical to numerical data). Lastly, Anna selects and reorders the variables required by a given classifier and passes a classifier-specific data frame to the classifier module by serializing the data to JSON and sending it with POST requests.

#### ML classifier prediction and results storage

**Fig. 5:**
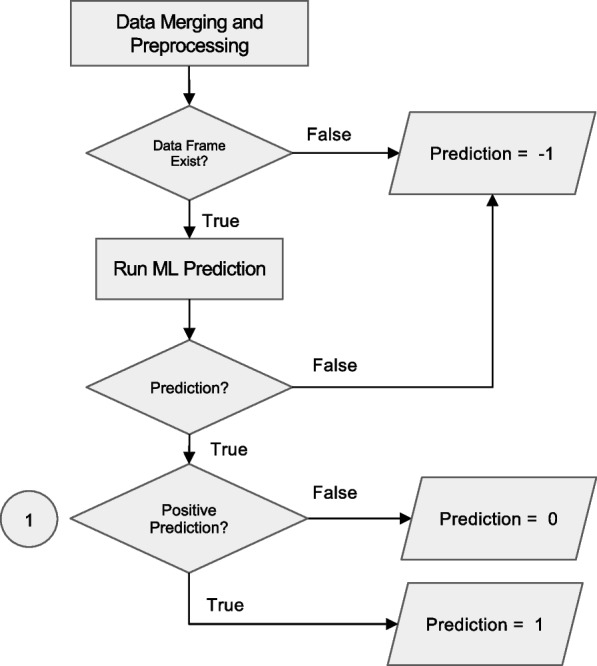
Workflow for prediction handling and result routing in the Anna platform. After data merging and preprocessing, the system checks for the existence of a valid data frame. If data is insufficient, a code of −1 is assigned and logged. If valid, a machine learning (ML) prediction is attempted. Programmatic errors during execution result in a code of −2, which is also logged. Successful predictions are evaluated for positivity: negative results are coded as 0 and positive as 1. All valid predictions (0 or 1) are written to the SQL database for downstream integration and access

Classifier results are generated using a dedicated server for each classifier (Fig. 1). This setup allows for a distinct computing environment for each classifier while ensuring security by restricting incoming requests solely to the Anna core server. For every line of the data frame, i.e. each unique set of test combinations, the classifier server generates the following information: 1) the prediction result (0/1 for binary classifiers, multi-level labels for multi-class classifier, 2) the IDs of tests that were used to generate the result, and 3) a timestamp reflecting when the ML classifier was first run on this particular data (Fig. 5). This information is returned to the EHR database via JSON formatted string and stored in a MySQL database for legal purposes. Since classifier results might influence clinical decision making, they are considered part of the medical record and need to be archived. In the current implementation, changes to the EHR system are not possible, which requires storage within Anna.

#### Insufficient data and error reporting

Anna automatically attempts to run all available ML classifiers for every request. If there is insufficient data to run a given classifier, either because a required laboratory test was not performed or a required value is missing, Anna reports a “−1” result in the prediction field of the JSON string sent to the EHR system. If the classifier is triggered but exits unsuccessfully, a result of ‘−2’ is reported. In addition, Anna will store the error code and supporting information such as the start and end of a session, including intermediate steps with timestamps in log files locally. To identify each session, a random 6-digit ID is assigned so that Anna developers can quickly locate problems in the log files.

#### Results visualization

**Fig. 6:**
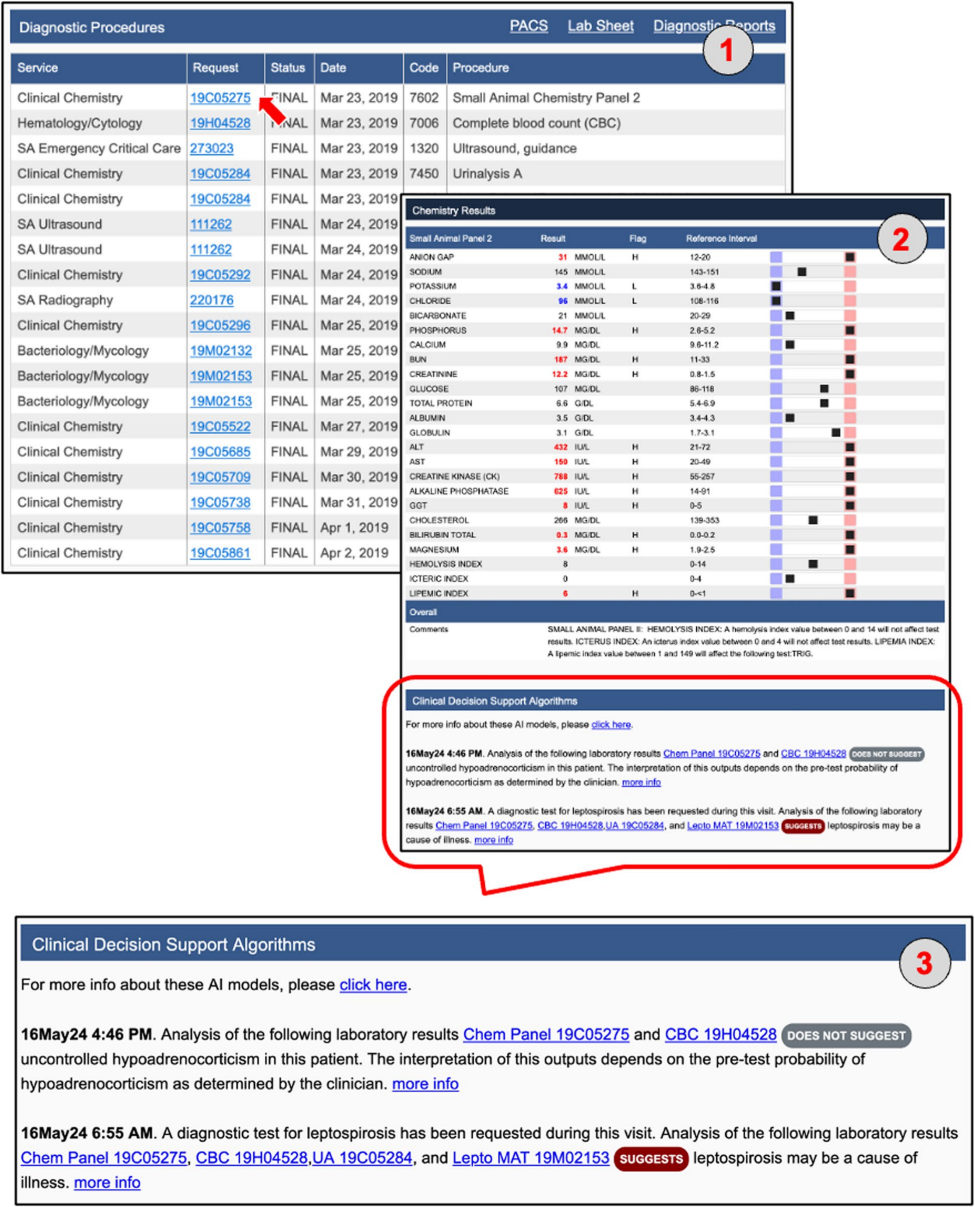
The Anna workflow from an end-user perspective. (1) Selecting a “Small Animal Panel 2” test in the “Diagnostic Procedures” section of the medical record (red arrow) opens the results page and automatically triggers Anna. The system identifies additional test results available within the predefined time window (see Fig. 2) and determines that the combined data are sufficient to generate predictions for the Addison’s disease and leptospirosis classifiers, but not for the liver shunt classifier. (2, 3) Prediction results for the available classifiers are displayed in the “Clinical Decision Support Algorithms” section at the bottom of the page. Test IDs contributing to each classifier’s output are presented as hyperlinks, allowing users to directly access the corresponding laboratory results. The timestamp indicates when each prediction was first generated, enabling users to assess whether the prediction result was available in time to inform clinical decision-making. In this example, both classifiers were implemented in 2024, indicating that the predictions would not have been available to the clinician managing the case in 2019. This time-stamping mechanism was introduced in part for legal reasons, to clearly distinguish retrospective predictions from those that were accessible during real-time clinical decision-making and to prevent misinterpretation of system capabilities at the time of care

The visualization of the JSON results string supplied by Anna needs to be implemented within the EHR system as Anna is purely a backend application. At UC Davis, we chose to present classifier results within a dedicated section titled *Clinical Decision Support Algorithms*, located at the bottom of each test result report that contributes input data to the model (Fig. 6). For instance, if a prediction is generated using CBC and chemistry panel data, the corresponding results will appear at the bottom of both the CBC and chemistry panel reports. While this reflects our implementation at Davis, other institutions may choose alternative formats that align with their clinical reporting practices. Since Anna automatically triggers all available classifiers for which the required input data is available, results from more than one classifier might be displayed. If none of the available classifiers renders a prediction, the EHR will display a message “There are no machine learning classifier results available for these data.”. A hyperlink to supplementary information about the specific ML classifier such as previously published performance metrics and interpretation guidelines is also provided to help the user understand the classifier’s purpose and functionality (Fig. 6).

### Classifier validation

In general, implementing ML classifiers in an environment outside the one in which they were originally developed requires careful re-validation to ensure their applicability to the new clinical setting. Differences in data distribution, laboratory instrumentation, or patient populations can lead to domain shifts that may impact model performance. In our case, all three integrated classifiers were originally developed at UC Davis, meaning that the environments for development/validation and real-world deployment were effectively identical, apart from the temporal offset between both. As such, our primary focus during the initial integration was to ensure computational fidelity: specifically, that the classifiers embedded within Anna produced identical outputs to those generated by the developers’ original implementation under the same conditions. To verify this, we requested a set of representative test cases from the model developers and compared Anna’s prediction outputs against the expected results. This confirmed that embedding a classifier into Anna did not alter its behavior. To ensure reliable classifier performance over time, we are implementing a prospective validation framework that compares Anna’s stored classifier predictions with finalized clinical diagnoses, once confirmatory diagnostics (e.g., imaging, specialist interpretation, or follow-up tests) are complete. This real-world evaluation will enable us to detect performance degradation over time due to domain shifts and support the continuous monitoring and re-calibration of embedded classifiers. Institutions intending to deploy Anna with locally developed or externally sourced models are advised to conduct similar validation steps, both prior to deployment and as part of ongoing clinical quality assurance.

### Anna security measures

Because Anna facilitates the exchange of sensitive clinical data between EHRs and ML classifiers, robust security measures are essential to ensure data integrity and prevent unauthorized access. To protect the Anna core server, incoming network traffic is controlled by two measures. First, Anna is accessible only within a specific network domain by routing through a virtual private network (VPN). Second, Anna authenticates the IP address of every incoming connection by cross-referencing the HTTP headers forwarded by the VPN service against an approved list of IP addresses. If an incoming IP does not match this list, the connection is terminated with a *403 Forbidden* status, indicating the request is understood but not authorized.

Furthermore, Anna developers can remotely control when EHR can communicate to each ML classifier’s computation server through the Anna core server. Anna will refuse to respond to EHR requests with a *503 Service Unavailable* status when the URL path is manually set to disabled.

**Fig. 7:**
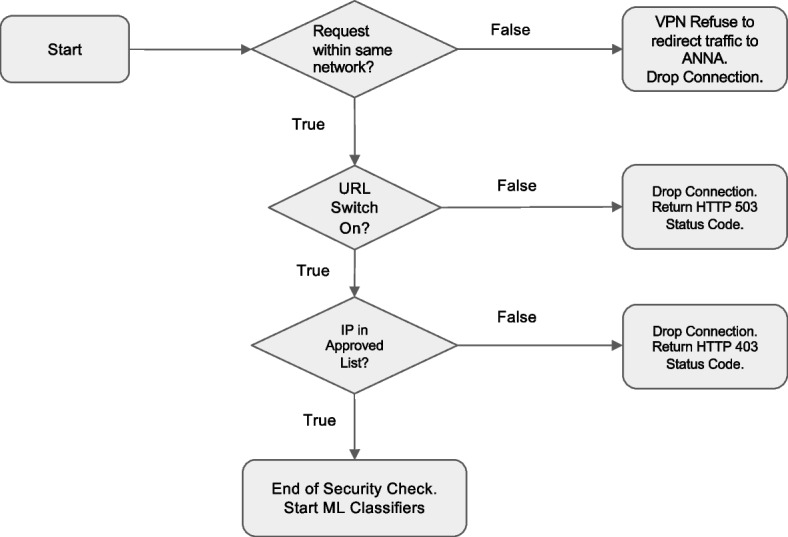
Decision logic of the measures of protecting Anna from unintended access

To ensure secure communications between the Anna core server and the ML classifier servers, the DNS for each classifier server is set to 127.0.0.1, known as “localhost” or “loopback address.” This keeps connections confined within the virtual machine. The Anna core server then makes local connections to each classifier server when receiving requests from the EHR system. Each classifier server is assigned a unique port number for distinction (Fig. 7).

### Integration of new classifiers

One of Anna's defining strengths is its ability to integrate new machine learning classifiers, regardless of the programming language in which they were developed. Because each classifier operates on a dedicated server, integrating new models does not interfere with existing ones. To maintain a consistent approach and format, we have developed a standard operating procedure (SOP) for integrating new classifiers. This SOP is available in the “resources” folder of our GitHub repository. It defines the structure of the source code and output data and outlines the procedures for incorporating, validating and deploying new classifiers into a production environment. The main steps of integrating a new classifier are as follows: 1) Obtain the required code, model files, validation data, and supporting information from the developer; 2) Define the decision logic and data preprocessing rules necessary for model execution; 3) Integrate the model into Anna and validate its output against the developers’ results; 4) Document any issues encountered during integration process and additional notes for record keeping; and 5) Release the updated version to the public and provide end-users with clear explanations of the newly added classifiers.

### Continuous performance monitoring

To monitor potential performance degradation over time, we have implemented a prospective validation strategy. At regular intervals (e.g., every six months), finalized clinical diagnoses are retrieved from the electronic health record (EHR) for all classifier predictions made during the corresponding period. These finalized diagnoses are treated as the ground truth. Concordance between the classifier's predictions and the final diagnoses is assessed using the large language model GPT-4o. Discordant cases are flagged for review by a subject matter expert. If the discrepancy is due to a misinterpretation by the language model, the output is corrected. Subsequently, performance metrics are computed. To ensure the reliability of the language model, a subset of its predictions is also reviewed manually. Because the review process identifies both true positive and true negative cases, the classifier can be periodically updated using these expert-validated cases.

## Results/discussion

Herein we describe Anna, an animal health analytics platform, that provides the computing infrastructure to integrate ML classifiers with EHR systems for real-time analysis of veterinary laboratory data.

Integrating ML classifiers into veterinary EHR systems can be challenging due to resource limitations, the rigidity of EHR systems or proprietary barriers. Since the UC Davis Veterinary Medical Teaching Hospital's EHR was developed in-house, modifications to the EHR were generally feasible, but they needed to be minor in scope and require minimal IT resources. To meet these constraints, we shifted classifier execution to a stand-alone platform and leveraged existing EHR reporting tools to enable data transfer between Anna and the EHR. EHR modifications were limited to minimal changes to the export formats for laboratory results, the addition of the Anna server IP as a trusted system and the display of classifier results. Commercial EHR systems commonly use closed architectures, which restrict the ability to modify workflows or incorporate external tools like Anna. Given the multitude of EHR systems in use and the potential legal, technical, and institutional barriers associated with each, it was beyond the scope of this study to systematically evaluate Anna’s compatibility across a range of platforms. Future work may explore strategies for broader integration, particularly within diverse clinical environments that rely on varying EHR infrastructures.

The use of open-source software as the Anna backbone reduces cost and enhances accessibility for the broader veterinary community [12, 13]. The rich support for multiple programming languages, third-party modules and plugins, and ability to reproduce in deployment helps Apache stand out as the ideal framework for hosting the backend server. Python's compatibility with different programming languages enables the integration of new ML classifiers, whether written in Python or not, without requiring significant code changes [14, 15]. Additionally, its modular design allows for easy maintenance and future expansions with minimal disruption, as each ML classifier runs on its own dedicated server. Since Anna and the EHR are two separate systems and architectures, errors occurring in Anna will not affect the EHR, and vice versa. This ensures the stability of the EHR system as it is, in contrast to Anna, essential to the users in veterinary practices and clinics.

Hosting ML classifiers on dedicated servers improves performance and ensures the forward compatibility of classifiers. Since Anna is designed to host a diverse array of machine learning classifiers, we needed to create suitable environments to accommodate various programming languages and platforms. When integrating the hypoadrenocorticism classifier into Anna, we initially encountered prolonged prediction times compared to running the classifier directly in MATLAB. This delay was primarily caused by Python initialization in the MATLAB runtime for every new request and could be resolved by a dedicated server running the ML classifier continuously. Implementing dedicated servers for ML classifiers also enables classifiers being run in legacy package versions with the dependencies used initially for compilation. This ensures the persistence of ML classifier results while keeping the Anna core server patched and updated. Independent servers can be easily set up in the existing environment without the need for additional hardware or software.

Anna simplifies obtaining predictions from multiple ML classifiers, overcoming the inefficiencies of current methods. Instead of visiting multiple sites for predictions or trying to set up classifiers locally, Anna fetches patient data directly from the EHR, runs the predictions on its backend server, and returns the results to the EHR for display. Users no longer need to manually input parameters or navigate different sites for various disease predictions, overcoming a major barrier for use in a clinical environment.

The visualization of classifier prediction results is done by the EHR system and requires minimal modifications. While Anna generates prediction results for all implemented classifiers and passes these on to the EHR using a standardized JSON string, it is up to the EHR administrators to decide how to display the prediction results within the EHR. In its current implementation at the UC Davis Veterinary Medical Teaching Hospital, classifier results are displayed at the bottom of all laboratory test results that were used as input for the prediction. For example, the Addison’s classifier results will be displayed with the complete blood count results and the chemistry panel results, which are the data that are used to generate the prediction. This provides users with quick access to classifiers results without having to navigate to another site. In addition, links to the specific laboratory tests that were used to make the prediction are provided.

Although veterinary medicine lacks data security laws like the Health Insurance Portability and Accountability Act (HIPAA) in human healthcare, securing patient data was a key focus in developing Anna. Healthcare data are sensitive since they are confidential [16] and usually come with personal identifiers that can be easily traced. Maintaining data privacy and preventing data leaks are two top concerns for adopting artificial intelligence into healthcare [17]. To address these concerns, Anna does not store patient data and relies on the EHR to control data access, keeping sensitive information secure. The only exception to this rule is the storage of classifier results, patient ID, test identifiers and timestamps in the open-source database management system MySQL. This became necessary because the classifier result is considered part of the medical record, but the EHR system of the Veterinary Medical Teaching Hospital at UC Davis does not yet provide a way to store this information. Yoon et. al introduced the concept of"accountability gap,"where using ML in clinical decisions can reduce human accountability, raising the question of responsibility for incorrect diagnoses [18]. As the adoption of AI into clinical practice becomes more commonplace, we anticipate that classifier results will be stored directly in the EHR rendering this functionality of Anna redundant.

Setting up Anna as a backend computational server offers three major benefits during development and deployment. First, separating Anna from the EHR reduces the risk of conflicts with existing EHR code when adding and testing new features during development. Since the EHR only handles presentation of the classifier results, fewer code changes are needed, and the risk of system crashes is lower compared to directly integrating ML classifiers into the EHR. Second, as a standalone server, Anna can be accessed by other EHR systems or authorized platforms without major code changes, thanks to its ability to control incoming traffic. Third, having separate teams working in parallel makes the integration progress smoother. This separation also allows for easier troubleshooting and maintenance, as issues related to Anna's operations can be resolved independently of the EHR system, minimizing disruptions to clinical workflows.

Compared to more feature-rich decision support platforms, Anna offers a simpler, locally hosted alternative that may be more suitable for veterinary settings with limited resources or stricter data governance requirements. There are currently no veterinary clinical decision support platforms that offer comparable functionality to Anna. In human healthcare, similar platforms include one developed by the University of California San Diego (UCSD) [19], and DEPLOYR [20] a system created by Standford University. Like Anna, both platforms can integrate clinical AI models, connect to existing EHR systems and return model results in real-time. However, in contrast to DEPLOYR (Microsoft Azure) and the UCSD system (Amazon Web Services), Anna is not dependent on cloud services for result generation and data storage. This design allows Anna to be deployed in environments with limited IT infrastructure, reduces ongoing operational costs, and enhances data privacy by keeping all computations and storage within institutional networks. Other advantages include ease of integration with existing EHRs, low cost, and complete local control over data, reducing both infrastructure complexity and privacy concerns. This makes Anna well-suited for academic, veterinary, or resource-limited environments.

However, compared to more mature cloud-based systems, Anna currently lacks advanced features such as automated performance monitoring, real-time drift detection, formal model versioning, and continuous integration/deployment (CI/CD) pipelines. While such tools are increasingly standard in human healthcare, their implementation in veterinary settings remains uncommon due to resource constraints and limited technical infrastructure. As Anna continues to evolve, future iterations will explore lightweight, scalable approaches to lifecycle management such as basic monitoring dashboards, periodic model audits, and version control strategies that are compatible with the practical realities of veterinary institutions.

Due to its broader adoption in veterinary medicine and the need to prioritize simplicity, a REST-based API was chosen over a more complex interoperability standard. Interoperability standards such as HL7 FHIR (Health Level Seven International Fast Healthcare Interoperability Resources) are important for facilitating scalable integration with external systems in human healthcare. However, HL7 FHIR has not been widely adopted in veterinary medical informatics, and most commercial veterinary EMRs do not support FHIR-based interfaces. Given these practical constraints and the limited development resources available for this proof-of-concept implementation, we prioritized core functionality and clinical integration over early adoption of emerging data standards. As the veterinary EMR ecosystem evolves, we anticipate integrating HL7 FHIR support using community-standard libraries such as HAPI HL7, thereby enhancing interoperability with emerging tools and systems.

The responsible adoption of AI in a veterinary teaching institution faces institutional and educational barriers and requires a phased implementation strategy. The lack of a defined institutional strategy for AI integration, especially with respect to the use of AI in a clinical setting, and limited AI literacy among faculty and students currently constrain broad deployment of Anna. To address these limitations, a phased implementation strategy has been designed to ensure safe, effective, and educationally integrated adoption. This includes (1) asynchronous training modules for clinicians and residents focused on AI literacy, model interpretation, and responsible clinical application, and (2) structured evaluation of user trust, perceived utility, and usability using repeated surveys based on the Technology Acceptance Model. The AI predictions will initially be accessible only to faculty and residents, with access contingent on completion of training. Feedback from this initial rollout will inform iterative improvements and future expansion to student users, with the long-term goal of embedding responsible AI use into both clinical care and veterinary education.

## Conclusions

With the rapid expansion of artificial intelligence in veterinary medicine, the need for centralized, secure, and fast ML servers integrated with EHR systems is becoming increasingly urgent. Such integration reduces data retrieval times, minimizes human errors, and streamlines access to various ML classifiers. This paper discusses the development of Anna, a stand-alone centralized backend machine learning platform that facilitates real-time data analysis with ML classifiers, aiding in clinical decision-making. Future iterations of Anna will focus on integrating more third-party classifiers, expanding its capabilities to handle time-series patient data, enabling interactive visualizations, generating predictions from multi-visit data, and becoming publicly accessible for users to upload their own data for machine learning predictions.

## Availability and requirements

Project name: Anna/UC Davis Animal Health Analytics Platform

Project home page: https://vmacs-analytics.vetmed.ucdavis.edu

Operating system(s): Platform independent

Programming language: Python

Other requirements: Apache, MATLAB, MySQL

License: GNU Affero General Public License

Any restrictions to use by non-academics: As defined by GNU Affero General Public License

## Supplementary Information

Supplementary Material 1. Supplementary Table 1: Table of ML Classifiers published to aid clinical-decision making in veterinary medicine

## Data Availability

This study did not utilize data sets. All code is available through https://github.com/ucdavis/ANNA-AnimalHealthAnalytics.

## Abbreviations

Anna: Animal Health Analytics Platform
JSON: JavaScript Object Notation
XML: Extensible Markup Language
EHR: Electronic Health Record
REST API: Representational State Transfer Application Programming Interface
HTTP: Hypertext Transfer Protocol
IP Address: Internet Protocol Address
VPN: Virtual Private Network
SQL: Structured Query Language
I/O bound: Input/Output bound
HL7: Health Level Seven International
FHIR: Fast Healthcare Interoperability Resources
HAPI: HL7 application programming interface
