# Supplementary material for: Anna: an open-source platform for real-time integration of machine learning classifiers with veterinary electronic health records
Source: BMC Vet Res. 2025 Oct 2;21:557. doi: 10.1186/s12917-025-05000-7 (PMC12492907; doi:10.1186/s12917-025-05000-7)
Supplement: Supplementary file 1 — Supplementary Material 1. Supplementary Table 1: Table of ML Classifiers published to aid clinical-decision making in veterinary medicine [file 12917_2025_5000_MOESM1_ESM.docx]

**Supplementary Materials**

| Classifiers | Species | Developers | GUI? | URL |
| --- | --- | --- | --- | --- |
| Lepto Classifier | Dogs | Department of Veterinary Medicine and Epidemiology, University of California, Davis, CA 95616, USA | Yes | https://vmacs-analytics.vetmed.ucdavis.edu/ml_classifier_run/leptospirosis |
| Addison’s Classifier | Dogs | Department of Veterinary Medicine and Epidemiology, University of California, Davis, CA 95616, USA | Yes | https://vmacs-analytics.vetmed.ucdavis.edu/ml_classifier_run/tommy_addisons |
| Shunt Classifier | Dogs | Department of Veterinary Medicine and Epidemiology, University of California, Davis, CA 95616, USA | Yes | https://vmacs-analytics.vetmed.ucdavis.edu/ml_classifier_run/shunt |
| Machine-learning based prediction of Cushing’s syndrome | Dogs | Pathobiology and Population Sciences, The Royal Veterinary College, Hawkshead Lane, North Mymms, Hatfield, AL9 7TA Herts UK | Unknown | NA |
| Field identification of infectious and inflammatory disorders of the central nervous system in cattle | Cattle | Department of Veterinary Sciences, University of Turin, Italy | Yes | https://cnsprediction.streamlit.app/ |
| Myxomatous Mitral Valve Disease | Dogs | Department of Veterinary Internal Medicine, College of Veterinary Medicine, Seoul, Republic of Korea | No | NA |

**Supplementary Table 1. Table of ML Classifiers published to aid clinical-decision making in veterinary medicine.**
